# One-year prospective real-world assessment of effectiveness and safety of erenumab in migraine prevention: results of the French FHU INOVPAIN registry study

**DOI:** 10.1186/s10194-023-01680-4

**Published:** 2023-11-08

**Authors:** M. Lanteri-Minet, R. Fabre, C. Martin, K. Pradat, A. Alchaar, E. Bozzolo, M. L. Duchene, E. K. Van Obberghen, A. Donnet, D. Fontaine

**Affiliations:** 1 Pain Department and FHU InovPain, CHU Nice and Côte Azur University, Hôpital Cimiez, 4 Rue Reine Victoria, 06003 Nice, France; 2 INSERM U1107 Migraine and Trigeminal Pain, Auvergne University, Clermont-Ferrand, France; 3 Public Health Department, CHU Nice and Côte Azur University, Nice, France; 4 Cinical Pharmacy Departement, CHU Nice and Côte Azur University, Nice, France; 5 Pain Departement, Timone Hospital, APHM, Marseille, France; 6 Neurosurgery Department and FHU InovPain, CHU Nice and Côte Azur University, Nice, France

**Keywords:** High frequency episodic migraine, Chronic migraine, Medication overuse, Erenumab, CGRP, Monoclonal antibodies, Real-world, France

## Abstract

**Background:**

Randomized clinical trials have demonstrated efficacy and safety of erenumab. The aim of this study is to evaluate the effectiveness and safety of erenumab in a real-world setting in French patients with migraine associated with extreme unmet needs.

**Methods:**

This is a one year-prospective real-word study with enrolment of all consecutive adult patients included in the FHU InovPain registry who participated in a compassionate erenumab use program.

**Results:**

Of 144 patients included, 140 patients (82.1% female / mean age of 50.9 ± 11.4) received at least one dose of erenumab and were concerned by effectiveness and safety assessment. All patients had failed 11 oral preventive treatments. Most of them suffered from chronic migraine (88.6%) and presented a medication overuse (90.7%) at baseline. Thirty-eight (27.1%) discontinued treatment during the 12-month follow-up, with 22 (15.7%), 11 (7.9%) and 5 (3.6%) patients before 3, 6 or 9 months of treatment. The proportion of ≥ 50% responders at M3, M6, M9 and M12 was 74/140 (52.9%), 69/118 (58.5%), 61/107 (57.0%) and 60/102 (58.8%) respectively. At M3, the rate of reversion from chronic migraine to episodic migraine was 57.3% and the rate of transition from medication overuse to non-overuse was 46.5%. For monthly migraine days, the median (IQR) was 18.0 (13.0–26.0), 9.0 (5.0–17.0), 7.5 (5.0–14.0), 8.0 (5.0–12.5) and 8.0 (5.0–12.0) at M0, M3, M6, M9 and M12 respectively. For HIT-6 score, the median (IQR) was 68.0 (63.8–73.3), 60.0 (54.0–65.0), 60.0 (50.3–53.0), 59.0 (50.0–63.0) and 58.0 (50.0–62.9) at M0, M3, M6, M9 and M12 respectively. Fifty-three (37.9%) patients reported at least one of the following adverse events: cutaneous erythema and/or pain at the injection site for 42 (30%) patients, constipation for 22 (15.7%) patients, muscle spasm for 2 (1.4%) patients, alopecia for one (0.7%) patient and blood pressure increase in one (0.7%) patient. There was no serious adverse event. One female patient became pregnant after 5 months of exposure to erenumab with a safe evolution after treatment discontinuation.

**Conclusion:**

This first French real-world study related to migraine prevention with CGRP-mAbs confirms effectiveness and safety of erenumab in patients with extreme unmet needs.

## Background

Targeting the pathway of calcitonin gene-related peptide (CGRP) has opened a new era for migraine treatment [1]. Thus, the prophylaxis of migraine has experienced major changes since the introduction of monoclonal antibodies (mAbs) targeting the CGRP (eptinezumab, galcanezumab, fremanezumab) or the CGRP receptor (erenumab), together referred to as CGRP-mAbs. These substances form the first class of drugs specifically developed for migraine prevention and randomized clinical trials have demonstrated both their efficacy [2] and short-term safety [3]. Altogether, CGRP-mAbs are approved for migraine with at least 4 migraine days per month but access and reimbursement differ from one country to to another, despite an evaluation based on the same evidence and in particular on randomized clinical trials that involved difficult-to-treat patients with multiple treatment failures [4–7]. In France, the Transparency Committee (*Commission de Transparence*) of the French National Authority for Health (*Haute Autorité de Santé*) has approved the principle of the reimbursement of CGRP-mAbs for the target population of migraine sufferers with at least 8 migraine days per month and at least two previous oral prophylactic treatment failures. However, due to the absence of direct efficacy comparison with a clinically relevant comparator, the Transparency Committee rated the clinical added value (CAV) of CGRP-mAbs at level V. With a CAV level V,meaning no improvement with respect to therapeutic strategy, it was not possible to reach an agreement following price negotiations between the pharmaceutical companies and the Economic and Public Health Evaluation Committee (*Comité Economique des Produits de Santé*). As a result, CGRP-mAbs are not reimbursed for French migraine sufferers. Two antibodies (fremanezumab and galcanezumab) are available on an ‘out of pocket’ basis and a third (eptinezumab) is in the process of being integrated into the treatments offered by certain hospitals (this integration is subject to the medico-economic evaluation carried out by each hospital).

In this context of limited access to CGRP-mAbs, Novartis Pharma SAS has setup a compassionate use program in order to make erenumab available free of charge for one year to adult migraine patients who are in need of prophylaxis and for whom no acceptable alternative prophylactic treatment was available. This compasionate program was accessible to French Neurologists under their full responsibility and this has enabled us to offer cost-free erenumab to the most severe patients followed in our center. This opportunity was all the more important for these patients as onabotulinumtoxinA was still not approved for the prophylaxis of chronic migraine by the French health authorities when the compassionate program was initiated. As well as responding to the unmet needs of our most severely affected patients, this has provided an opportunity to set up the first French real-word study on CGRP-mAbs in migraine prevention.

## Methods

### Research context

This study has been carried out as part of the *Federation Hospitalo-Universitaire* (FHU) InovPain. FHU InovPain consists of a research network dedicated to innovative solutions for chronic refractory pain, including headache disorders. This network brings together a number of research teams from public institutions and private healthcare companies, under the coordination of the Côte Azur University and the Nice University Hospital. Approved by the National Alliance for Life Sciences and Health (AVIESAN), it is currently the only FHU dedicated to pain and headaches in France. Like all FHUs in France, this network was built to facilitate translational research in all its facets from basic research, translation to humans, translation to patients, translation to clinical practice and translation to the community. With regard to these last two aspects, which involve clinical implementation and public health, FHU InovPain is based on a patient registry to facilitate real-world observational research and, in particular, to assist French health authorities and manufacturers to collect data for the assessment of new drugs and/or new medical devices before and after they are marketed.

### Type of study

This study is a one year-prospective, cohort, real-word study carried out in the Pain Department of the Nice University Hospital.

### Participants

All consecutive adult patients included in the FHU InovPain registry who participated in the compassionate erenumab use program set up by Novartis Pharma SAS were considered for enrolment. The inclusion criteria for the compassionate erenumab use program were: i) age over 18 years; ii) migraine (without or with aura) according to the diagnostic criteria of the 3rd edition of the International Classification of Headache Disorders (ICHD-3); iii) at least 8 migraine days per month confirmed in a headache diary kept prospectively for at least one month before starting the program; iv) impact requiring prophylactic treatment according to the practitioner's judgement and v) no therapeutic alternative to the prescription of a CGRP-mAb. This last inclusion criterion corresponded to failure (efficacy failure after administration of drug for at least 2–3 months at generally accepted therapeutic doses / tolerability failure defined as discontinuation due to adverse event / contraindication) for all treatments with an approval for migraine prevention (amitriptyline, flunarizine, metoprolol, oxetorone, pizotifen, propranolol, topiramate) and those used off-label in tertiary migraine treatment centers (candesartan, lisinopril, sodium valproate, venlafaxine). OnabotulinumtoxinA was not considered in this last criterion because it was still not approved for the prophylaxis of chronic migraine by the French health authorities when patients were included in the compassionate erenumab use program. The exclusion criteria for the compassionate erenumab use program were: i) hypersensitivity to the active substance or to one of the excipients; ii) pregnant or breast-feeding woman; and iii) history of cardiovascular disease. The last exclusion criterion did not concern high blood pressure (HBP), provided that it was well controlled by antihypertensive treatment.

### Treatment

Erenumab was administered every 4 weeks (± 4 days), the subcutaneous injection (thigh, abdomen or arm) being carried out by a nurse or physician at the Nice University Hospital, to which the patient came monthly for his/her assessment and treatment visit. The treatment was available for one year, with a maximum of 12 injections under this compassionate program. The unit dose was 70 mg or 140 mg at the start of treatment, depending on the practitioner's judgement of the severity of the patient's clinical condition. If the unit dose at M0 was 70 mg and was well tolerated, it could be increased to 140 mg if the therapeutic effect was deemed insufficient. At M3, treatment was continued if the patient showed a reduction in migraine days of at least 30%. Despite a reduction in migraine days of less than 30%, treatment could be continued if headache days and/or drug consumption had been reduced by at least 30% and the practitioner judged these reductions to be clinically relevant. After the first two administrations of erenumab (M0 and M1) and throughout the compassionate erenumab use program, the unit dose remained flexible (possibility of increasing to 140 or reducing to 70 mg) depending on the effectiveness and tolerability assessment after discussion between the patient and the practitioner.

### Assessment

Assessment was performed at baseline and each month by a trained neurologist with a face-to-face interview using the file that allowed to prospectively collect data in the FHU InovPain registry. This file included a headache diary filled in prospectively to assess: i) the monthly number of headache days (MHD); ii) the monthly number of migraine days (MMD) (defined as a day with a headache meeting the ICHD-3 C and D criteria for migraine without aura, or a day with a symptomatology meeting the ICHD-3 B and C criteria for migraine with aura, or one day with a headache considered by the patient to be migraine at onset and relieved by taking a specific migraine medication) and iii) the monthly number of days with acute treatment and/or symptomatic headache treatment used to assess the presence of medication overuse (MO) defined according to ICHD-3 criteria: ≥ 10 days per month of consumption for triptans, opioids and drug combinations; ≥ 15 days per month of consumption for paracetamol, aspirin and non-steroidal anti-inflammatory drugs. This file also included various questionnaires patient-reported outcome (PRO) measures such as the HAD scale to assess emotional impairment (anxiety and depression), the HIT-6 scale to assess impact, the EQ5-D scale to assess health status and the Patient Global Impression of Change (PGIC). For this study, the data considered were: i) MHD and MMD in the month preceding the first administration of erenumab and then each month for one year (or until study discontinuation for patients not receiving one year's treatment) in order to establish the mean MHD and MMD at baseline (M0) and at 3 (M3), 6 (M6), 9 (M9) and 12 (M12) months following the first erenumab administration; ii) migraine form (high frequency episodic migraine [HFEM] or chronic migraine [CM]) at baseline (M0) and at 3 (M3), 6 (M6), 9 (M9) and 12 (M12) months following the first erenumab administration; iii) presence or absence of MO before the first administration of erenumab (M0) and at 3 (M3), 6 (M6), 9 (M9) and 12 (M12) months following this first administration; iv) HAD anxiety score and HAD depression score before the first administration of erenumab (M0); v) EQ-5 index before the first administration of erenumab (M0); vi) HIT-6 score before the first administration of erenumab (M0) and at 3 (M3), 6 (M6), 9 (M9) and 12 (M12) months following this first administration; vii) PGIC score at 3 (M3), 6 (M6), 9 (M9) and 12 (M12) months following the first administration of erenumab. Adverse events were also recorded at each monthly visit to assess and administer the treatment. Safety data were collected by questioning by a nurse or doctor and by systematically taking blood pressure.

### Outcomes

The primary efficacy endpoint was the proportion of patients who achieved at least 50% reduction from baseline (M0) in MMDs at M3, M6, M9 and M12. Secondary efficacy endpoints were the change from baseline (M0) in MMDs, MHDs and HIT-6 at M3, M6, M9 and M12. The following secondary endpoints were also assessed: proportion of patients who achieved at least 75% reduction from baseline (M0) in MMDs at M3, M6, M9 and M12, proportion of patients who achieved at least 30% reduction from baseline (M0) in MMDs at M3, M6, M9 and M12, proportion of patients converting from CM at baseline to EM at M3, M6, M9 and M12, proportion of patients converting from MO at baseline to non-MO at M3, M6, M9 and M12, PGIC at M3, M6, M9 and M12. Safety and tolerability were assessed by observed or reported adverse events.

### Statistics

The study’s sample size was not calculated based on statistical considerations. All consecutive adult patients included in the FHU InovPain registry who participated to the compassionate erenumab use program set up by Novartis Pharma SAS from April 2019 to September 2021 were included.

The data were described using the mean and standard deviation [SD] or median interquartile range [IQR] for quantitative variables and the frequency and percentage for qualitative variables. To address the primary endpoint, the 50% responder rate was studied. Data at M6, M9 and M12 were compared with those obtained at M3 using McNemar tests. As 3 comparisons were performed for each measure, a *p*-value < 0.016 (0.05/3) was considered as significant. Data collected at baseline (age, gender, MMD, MHD, migraine form, MO, HAD A, HAD D, EQ-5D, HIT-6, psychiatric comorbidities, others comorbidities, tripan responsiveness) were tested using univariate analysis to determine if they were significantly related to the 50% responder rate at M3, M6, M9 and M12. Student's t-tests were used for quantitative variables and Chi-square tests for qualitative variables. Variables with *p*-value ≤ 0.05 in univariate analysis were used to construct multivariate logistic regression. Only variables with *p*-value ≤ 0.05 in the final model were kept. Adjusted Odds-ratio (AdjOR) and 95% confidence intervals (95%CI) were shown. In order to compare changes in MMD, MHD, HIT score, the presence of MO and the migraine form between baseline and the different assessment times (M3, M6, M9 and M12), paired Student's t-tests (quantitative variables) and McNemar tests (qualitative variables) were performed. As 4 comparisons were performed for each measure, a *p*-value < 0.013 (0.05/4) was considered as significant. Change from baseline to M3, M6, M9 and M12 for MMD, MHD and HIT scores were calculated. Due to the discontinuation of erenumab by some patients prior to M12, additional analysis of responder rate, MMDs and MHDs was performed using a last observation carried forward (LOCF) analysis. Analyses were performed using R-4.3.0 software.

### Ethics / regulations

This observational study did not require the authorization of an ethics committee. This study was part of the FHU InovPain registry clinical research program which received authorization from the National Commission on Informatics and Liberty (*Commission Nationale de l’Information et des Libertés—CNIL* / CIL register no. 278 dated 11/09/2017). Patients were included only after signing two separate written informed consents: a first one enabling their personal data to be transmitted to Novartis Pharma SAS to validate their eligibility in the compassionate program allowing them to benefit from free erenumab and a second allowing the data collected in the FHU InovPain registry database to be used for clinical research purposes.

The study was performed according to the Strengthening the Reporting of Observational Studies in Epidemiology (STROBE) guidelines [8]. Novartis was involved only in the supply of erenumab to our hospital pharmacy without direct contact with patients included in the study. Novartis was not involved in the collection, quality control and analysis of the data.

## Results

### Demographic and headache characteristics at baseline

**Fig. 1:**
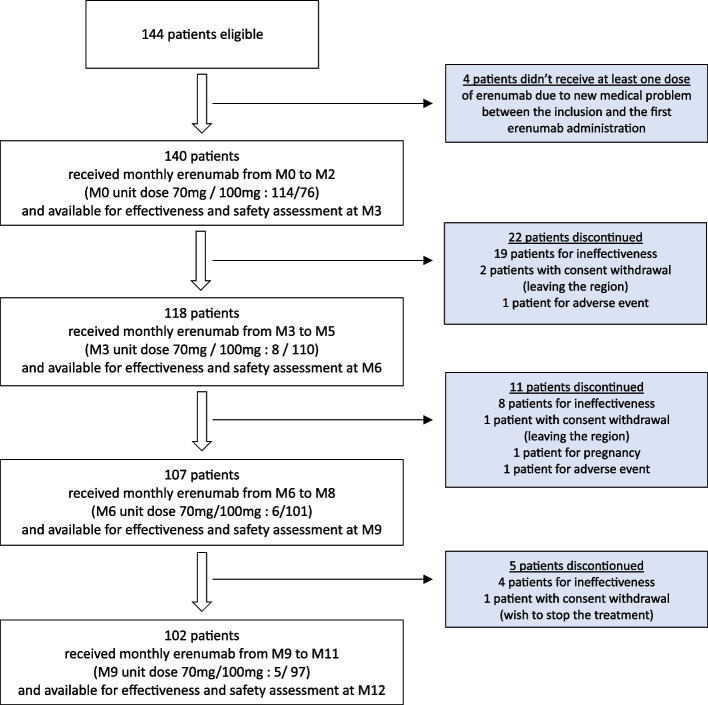
Flow-chart of patients from inclusion to study end with initial evaluation at baseline (M0) and evaluation at month 3 (M3), month 6 (M6), month 9 (M9) and month 12 (M12)

One hundred and forty-four patients were included in the study. One hundred and forty patients received at least one dose of erenumab and were concerned by effectiveness and safety assessment. One hundred and forty, 118, 107 and 102 patients were available for effectiveness and safety assessment at M3, M6, M9 and M12, respectively. Reasons for study discontinuation are reported in Fig. 1. Thirty-eight (27.1%) discontinued treatment during the 12-month follow-up, with 22 (15.7%), 11 (7.9%) and 5 (3.6%) patients before 3, 6 or 9 months of treatment respectively. The major cause of discontinuation was ineffectiveness (*n* = 31/38; 81.5%) followed by withdrawal because of patient’s choice (*n* = 4/38; 10.5%), adverse event (*n* = 2/38; 5.3%) and pregnancy (*n* = 1/38; 2.6%). The majority of patients who received at least one dose of erenumab and concerned by assessment were female (82.1%) with a mean age of 50.9 ± 11.4 (range: 19–75). Demographic and headache baseline characteristics of patients are reported in Table 1. Most of the patients (88.6%) suffered from CM. Most of the patients (90.7%) presented with a baseline MO which involved mainly nonsteroidal anti-inflammatory drugs (22.9% of the included population), weak opioids (25.7% of the included population) and triptans (71.4% of the included population). According to inclusion criteria of the compassionate erenumab use program, all patients had failed 11 oral preventive treatments and only 4 (2.9%) had continued oral prophylactic treatment.

**Table 1:** Demographic and headache baseline characteristics of patients who received at least one dose of erenumab and concerned by effectiveness and safety assessment (*n* = 140)

Characteristics	*N* = 140
Gender	
Female	115 (82.1%)
Male	25 (17.9%)
Age	50.9 ± 11.4
MHDs	22.5 ± 7.3
MMDs	19.6 ± 7.4
Migraine form	
MEHF	16 (11.4%)
CM	124 (88.6%)
Medication overuse	
Yes	127 (90.7%)
No	13 (9.3%)
HAD	
Anxiety	9.5 ± 4.4
Depression	7.5 ± 4.8
HIT-6	68.0 ± 5.9
EQ-5D	0.55 ± 0.30
Concurrent oral preventive treatment	
Yes	4 (2.9%)
No	136 (97.1%)
Psychiatric comorbidities	
Yes	69 (49.3%)
No	71 (50.7%)
Other comorbidities	
Yes	38 (27.1%)
No	102 (72.9%)
Triptan responsiveness	
Yes	106 (75.7%)
No	34 (24.3%)

### Unit dose throughout the compassionate erenumab use program

The repartition of erenumab unit dose (70 mg / 140 mg) was 114 / 26, 13 / 127, 8 / 110, 6 /101, 5 / 97 and 5 / 97 at M0, M1, M3, M6, M9 and M12 respectively (Fig. 1).

### Primary efficacy endpoint

**Fig. 2:**
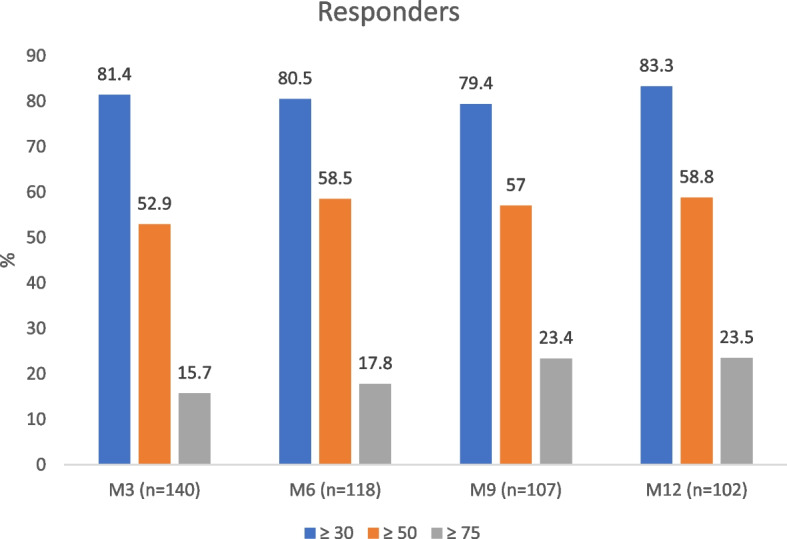
Reponder rates (≥ 30%, ≥ 50% as primary endopoint, ≥ 75%) et M3, M6, M9 and M12

The proportion of ≥ 50% responders at M3, M6, M9 and M12 was 74/140 (52.9%), 69/118 (58.5%), 61/107 (57.0%) and 60/102 (58.8%) respectively (Fig. 2). Comparison of responder rates at M6, M9 and M12 with those at M3 showed no statistically significant difference (*p* = 0.505, *p* = 0.330 and *p* = 0.493 respectively). In univariate analysis, a positive association emerged between the 50% response rate at M3 and MMD (*p* < 0.001), MHD (*p* < 0.001) and EQ-5D (*p* = 0.025) at baseline (Table 2). At M6, a positive association appeared between the 50% response rate and age (*p* = 0.039), MHD (*p* = 0.011), MMD (*p* = 0.001) and psychiatric comorbidities (*p* = 0.012) (Table 2). Only psychiatric comorbidities (*p* = 0.002) were associated with the 50% response rate at M9 (Table 2). Medication overuse (*p* = 0.039), EQ-5D (*p* = 0.012) and psychiatric comorbidities (*p* = 0.002) were associated with the 50% response rate at M12 (Table 2). Multivariate logistic regression developed from the variables with *p*-value ≤ 0.05 in univariate analysis identified baseline variables predictive of therapeutic response only for M6. Three variables found to be associated to the 50% responder rate at M6 were a lower MHD (AdjOR = 0.93, 95%CI = [0.89; 0.99], *p* = 0.036), a lower age (AdjOR = 0.96, 95%CI = [0.93; 0.99], *p* = 0.045) and absence of psychiatric comorbidities (AdjOR = 2.61, 95%CI = [1.19; 5.87], *p* = 0.017) were associated to the 50% responder rate at M6 (Table 3).

**Table 2:** Univariate analysis of independent determinants of ≥ 50% response at M3, M6, M9 and M12

	Responder at M3			Responder at M6			Responder at M9			Responder at M12		
	< 50%	≥ 50%	*p*-value	< 50%	≥ 50%	*p*-value	< 50%	≥ 50%	*p*-value	< 50%	≥ 50%	*p*-value
Gender (F/M; %)	47.0/48.0	53.0/52.0	0.925	39.2/52.4	60.8/47.6	0.266	43.2 ± 42.1	56.8 ± 57.9	0.931	42.9/33.3	57.1/66.7	0.456
Age, years (mean ± SD)	52.5 ± 11.8	49.5 ± 10.9	0.116	53.4 ± 12.0	49.0 ± 10.9	**0.039**	51.6 ± 12.0	50.2 ± 9.9	0.522	51.9 ± 12.0	50.1 ± 10.3	0.418
MHD at M0 (mean ± SD)	25.0 ± 6.6	20.3 ± 7.2	** < .001**	24.0 ± 6.7	20.6 ± 7.2	**0.011**	21.7 ± 7.1	21.5 ± 7.2	0.900	23.1 ± 6.7	20.7 ± 7.3	0.096
MMD at M0 (mean ± SD)	22.3 ± 7.3	17.2 ± 6.6	** < .001**	20.8 ± 7.0	17.3 ± 6.7	**0.001**	18.8 ± 6.7	17.9 6.9	0.455	19.2 ± 7.0	17.6 ± 6.5	0.223
Migraine form (CM/MEHF; %)	49.2/31.3	50.8/68.8	0.176	44.2/21.4	55.8/78.6	0.104	41.9/50.0	58.1/50.0	0.570	42.2/33.3	57.8/66.7	0.757
Medication overuse (Yes/No; %)	48.0/38.5	52.0/61.5	0.510	43.1/22.2	56.9/77.8	0.302	45.5/12.5	54.5/87.5	0.134	44.2/0.0	55.8/100.0	**0.039**
HAD A at M0 (mean ± SD)	10.0 ± 4.4	9.0 ± 4.4	0.194	9.9 ± 4.2	8.7 ± 4.5	0.147	9.6 ± 4.4	9.1 ± 4.3	0.541	9.6 ± 4.3	9.2 ± 4.5	0.670
HAD D at M0 (mean ± SD)	8.1 ± 4.9	6.9 ± 4.7	0.121	7.4 ± 5.4	6.9 ± 4.6	0.552	7.2 ± 5.0	6.9 ± 4.7	0.742	7.7 ± 5.1	6.6 ± 4.6	0.233
HIT-6 at M0 (mean ± SD)	69.0 ± 5.9	67.2 ± 5.9	0.075	68.4 ± 6.2	67.6 ± 6.0	0.471	68.5 ± 5.9	67.3 ± 6.2	0.314	68.5 ± 5.7	66.8 ± 6.0	0.144
EQ-5D at M0 (mean ± SD)	0.49 ± 0.31	0.60 ± 0.29	**0.025**	0.54 ± 0.32	0.59 ± 0.28	0.365	0.55 ± 0.29	0.58 ± 0.31	0.645	0.48 ± 0.31	0.63 ± 0.28	**0.012**
Psychiatric comorbidities (Yes/No; %)	52.2/42.3	47.8/57.7	0.240	53.6/30.6	46.4/69.4	**0.012**	58.8/28.6	41.2/71.4	**0.002**	57.1/26.4	42.9/73.6	**0.002**
Others comorbidities (Yes/No; %)	52.6/45.1	47.4/54.9	0.427	44.8/40.4	55.2/59.6	0.678	42.9/43.0	57.1/57.0	0.987	40.7/41.3	59.3/58.7	0.957
Triptan responsiveness (Yes/No; %)	45.3/52.9	54.7/47.1	0.436	40.2/46.2	59.8/53.8	0.587	43.0/42.9	53.0/57.1	0.989	40.7/42.9	59.3/57.1	0.861

**Table 3:** Logistic regression analysis of ≥ 50% reponse et M6

Variables	Coefficient (b)	SE	Wald χ2	*p*-value	Adj OR	[95% CI]
MHD	-0.06	0.03	4.0	0.036	0.93	[0.89; 0.99]
Age	-0.04	0.02	4.4	0.045	0.96	[0.93; 0.99]
Psychiatric comorbidities (No vs Yes)	0.96	0.40	5.6	0.017	2.61	[1.19; 5.87]

### Secondary efficacy endpoints

**Fig. 3:**
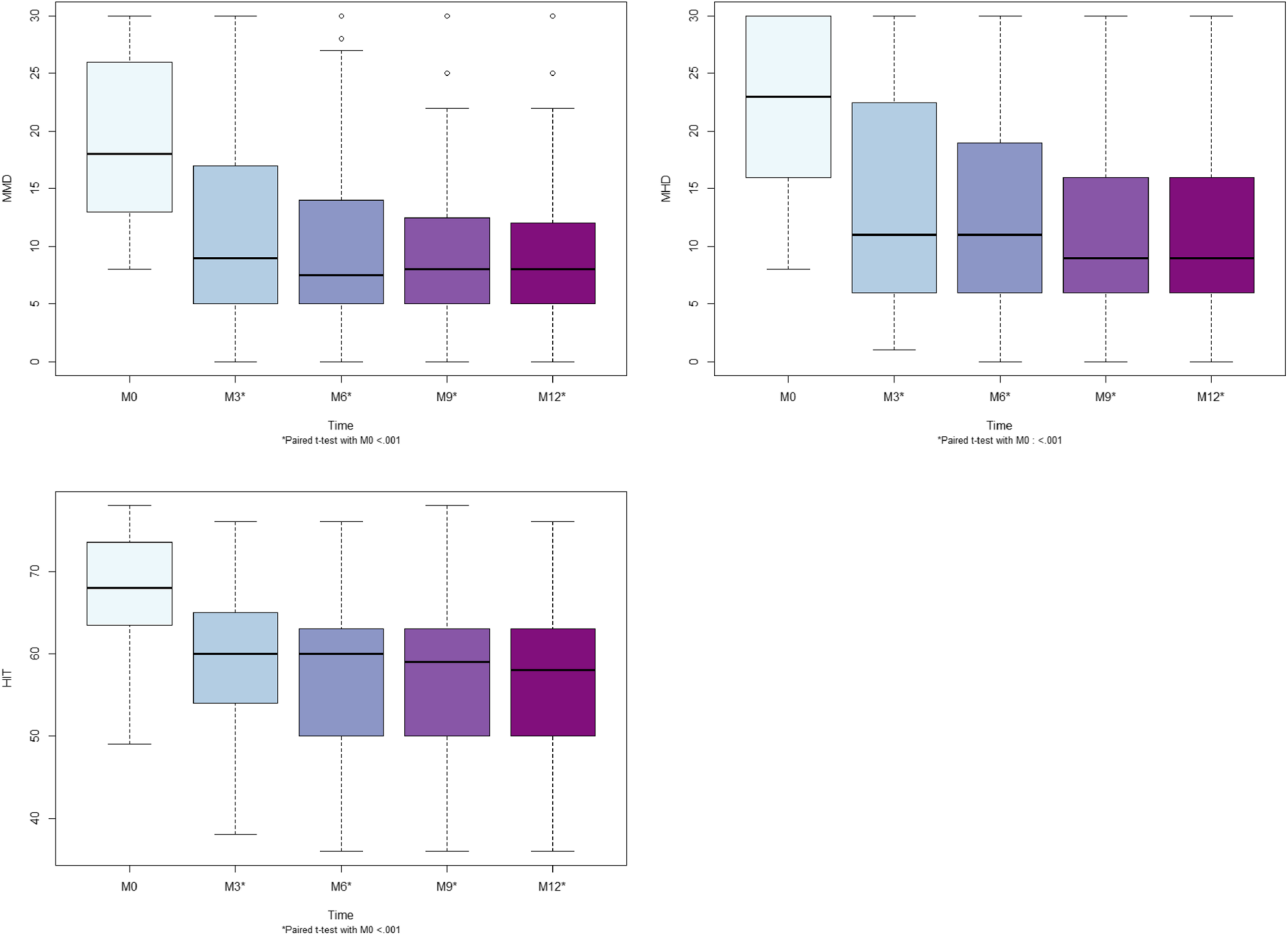
Median with IQR of monthly migriane days (MMDs), monthly headache days (MHDs) and HIT6-score (HIT) at M0, M3, M6, M9 and M12

For MMDs, the median (IQR) was 18.0 (13.0–26.0), 9.0 (5.0–17.0), 7.5 (5.0–14.0), 8.0 (5.0–12.5) and 8.0 (5.0–12.0) at M0, M3, M6, M9 and M12, respectively (Fig. 3). For MHDs, the median (IQR) was 23.0 (16.0–30.0), 11.0 (6.0–22.3), 11.0 (6.0–19.0), 9.0 (6.0–16.0) and 9.0 (6.0–15.8) at M0, M3, M6, M9 and M12, respectively (Fig. 3). For HIT-6 score, the median (IQR) was 68.0 (63.8–73.3), 60.0 (54.0–65.0), 60.0 (50.3–53.0), 59.0 (50.0–63.0) and 58.0 (50.0–62.9) at M0, M3, M6, M9 and M12, respectively (Fig. 3). For MMDs, MHDs and HIT-6 score, paired t-test of M3, M6, M9 and M12 with M0 showed statistically significant differences (*p* < 0.001). For MMDs, MHDs and HIT-6 score, changes from baseline (M0) at M3, M6, M9 and M12 are presented in Table 4.

**Table 4:** Monthly migriane days (MMDs), monthly headache days (MHDs) and HIT6-score (HIT) at M0, M3, M6, M9 and M12 with change from baseline (BL or M0) at M3, M6, M9 and M12

	M0	M3	M6	M9	M12
Patients (n)	140	140	118	107	102
MHDs	22.5 ± 7.3	14.5 ± 9.6^a^	13.4 ± 9.0^a^	12.2 ± 8.6^a^	11.9 ± 8.7^a^
*Change from BL*	-	8.0 ± 7.1	8.6 ± 7.4	9.4 ± 8.5	9.8 ± 7.8
MMDs	19.6 ± 7.4	11.5 ± 8.5^a^	10.0 ± 6.8^a^	9.2 ± 6.1^a^	9.0 ± 6.3^a^
*Change from BL*	-	8.1 ± 5.9	8.8 ± 6.1	9.1 ± 6.5	9.2 ± 5.7
HIT	68.0 ± 6.0	59.3 ± 8.1^a^	57.0 ± 8.9^a^	56.5 ± 9.9^a^	56.5 ± 9.0^a^
*Change from BL*	-	8.8 ± 7.7	10.9 ± 8.8	11.3 ± 9.6	11.1 ± 8.6

^a^Paired t-test with M0 < .001

The proportion of ≥ 75% responders at M3, M6, M9 and M12 was 22/140 (15.7%), 21/118 (17.8%), 25/107 (23.4%) and 24/102 (23.5%), respectively (Fig. 2). Comparison of 75% responder rates at M6, M9 and M12 with those at M3 showed no statistically significant difference (*p* = 1.000, *p* = 0.297 and *p* = 0.275 respectively). The proportion of ≥ 30% responders at M3, M6, M9 and M12 was 114/140 (81.4%), 95/118 (80.5%), 85/107 (79.4%) and 85/102 (83.3%) respectively (Fig. 2). Comparison of 30% responder rates at M6, M9 and M12 with those at M3 showed statistically significant differences (*p* = 0.001, *p* < 0.001 and *p* = 0.002 respectively).

**Fig. 4:**
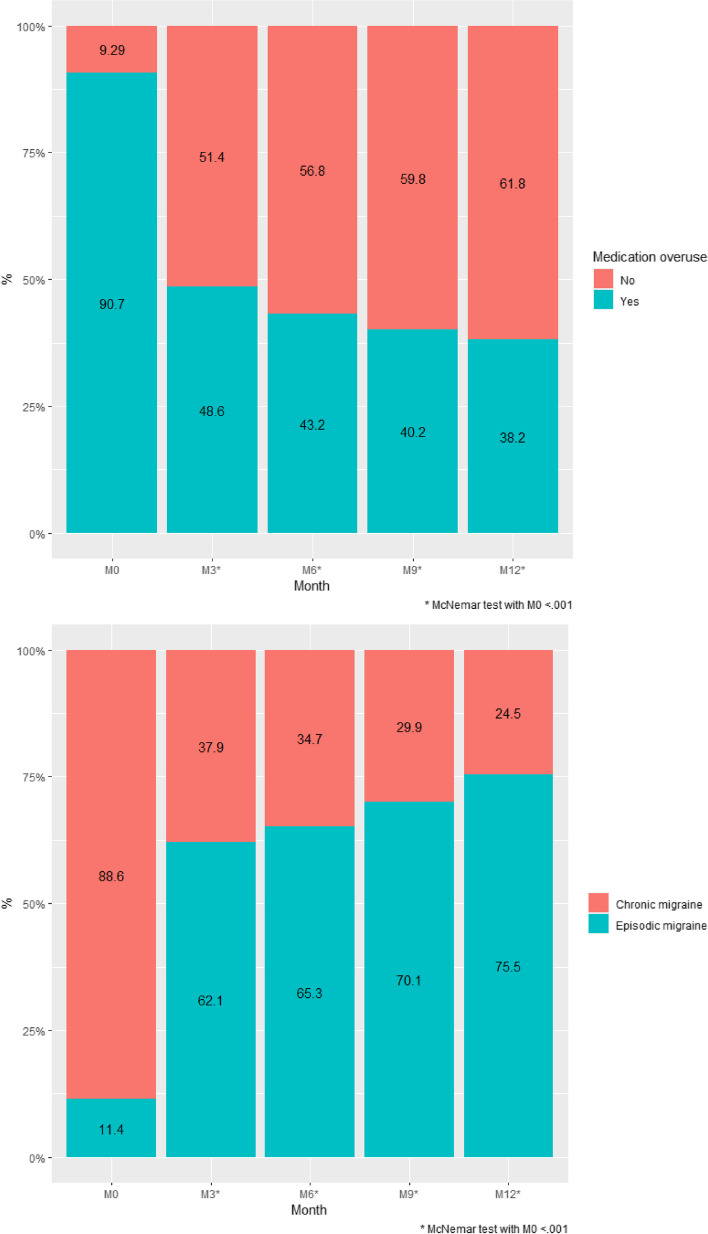
Proportion of medication overuse (MO) (yes/no) and migraine form (episodic/chronic) at M0, M3, M6, M9 and M12

The proportion of patients with CM was reduced at M3 (53/140, 37.9%), M6 (41/118, 34.7%), M9 (32/107, 29.9%) and M12 (25/102, 24.5%) compared with M0 (124/140, 88.6%) (*p* < 0.001) (Fig. 4). At M3, the rate of reversion from chronic migraine to episodic migraine was 57.3%. The proportion of patients with MO was reduced at M3 (68/140, 48.6%), M6 (51/118, 43.2%), M9 (43/107, 40.2%) and M12 (39/102, 38.2%) compared with M0 (127/140, 90.7%) (*p* < 0.001) (Fig. 4). At M3, the rate of transition from M0 to non-overuse was 46.5%

**Fig. 5:**
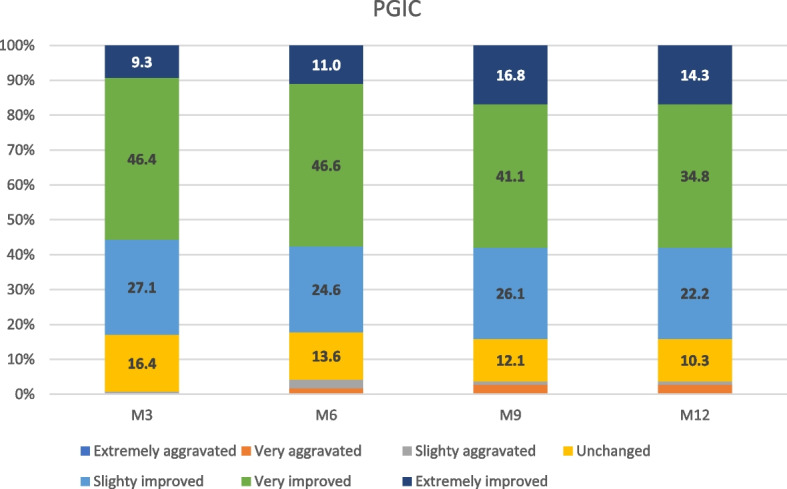
Patient Global Iimppression of Change (PGIC) at M3, M6, M9 and M12

PGIC at M3, M6, M9 and M12 are presented in Fig. 5. Patients who reported considerable or major improvement were 78/140 (55.7%), 68/118 (57.6%), 62/107 (57.9%) and 67/102 (65.7%) at M3, M6, M9 and M12, respectively.

### LOCF analysis

LOCF analysis showed similar results in terms of responder rates, MMDs, MHDs, HIT-6 score. Results are presented in Table 5.

**Table 5:** LOCF analysis

**Overall population (*N* = 140)**	M0	M3	M6	M9	M12
Response rate ≥ 30%—n (%)	-	114 (81.4)	98 (70.0)	92 (65.7)	93 (66.4)
Response rate ≥ 50%—n (%)	-	74 (52.9)	70 (50.0)	65 (46.4)	65 (46.4)
Response rate ≥ 75%—n (%)	-	22 (15.7)	22 (15.7)	27 (19.3)	27 (19.3)
MHDs	22.5 ± 7.3	14.5 ± 9.6*	15.1 ± 9.7*	14.9 ± 9.9*	15.0 ± 10.0*
*Change from BL*	-	8.0 ± 7.1	7.5 ± 7.5	7.6 ± 8.3	7.5 ± 7.9
MMDs	19.6 ± 7.4	11.5 ± 8.5*	12.0 ± 8.6*	12.2 ± 8.9*	12.3 ± 9.0*
*Change from BL*	-	8.1 ± 5.9	7.6 ± 6.4	7.5 ± 6.7	7.5 ± 7.9
HIT	68.0 ± 6.0	59.3 ± 8.1*	58.5 ± 9.3*	58.4 ± 10.1*	58.6 ± 9.4*
*Change from BL*	-	8.8 ± 7.7	9.6 ± 8.9	9.6 ± 9.6	9.4 ± 8.8

*Paired t-test with M0 <.001

### Safety and tolerability assessment

Fifty-three (37.9%) patients reported at least one adverse event including: cutaneous erythema and/or pain at the injection site for 42 (30%) patients, constipation for 22 (15.7%) patients, muscle spasm for 2 (1.4%) patients, alopecia for one (0.7%) patient and blood pressure increase in one (0.7%) patient. There was no serious adverse event. In two (1.4%) patients, constipation led to discontinuation of treatment at M3 in one patient and at M6 in the other. The patient who experienced an increase in blood pressure did so after 3 months of erenumab use. This patient had a history of well-controlled HBP at the time of inclusion in the compassionate erenumab use program. This worsening of HBP was judged by the patient's referring cardiologist to be of moderate intensity and was rapidly brought under control by adapting the antihypertensive treatment. In view of these elements and the fact that the concerned patient had a 75% response rate, it was decided that the benefit/risk ratio was in favor of erenumab and treatment was continued. No worsening of HBP was observed during the additional 9 months of the compassionate erenumab use program for this patient.

One female patient became pregnant after 5 months of exposure to erenumab. Treatment was stopped as soon as the pregnancy was known. The pregnancy and delivery proceeded normally with no neonatal problems.

## Discussion

A number of real-world studies of migraine treatment with CGRP-mAbs have been published with a majority of studies on erenumab which was the first to obtain approval for migraine prevention [9, 10]. These studies are characterized by a significant methodological heterogeneity [11] and interpretation of their results could be hampered by retrospective data collection, small patient numbers, and short follow-up periods. Nevertheless, some prospective studies, with at least one year's follow-up in more than 100 patients, support meaningful effectiveness and safety of erenumab in real-world patient populations including patients with CM, patients with MO and patients with multiple preventive treatment failures [12–15].

In this one year-prospective clinical-based study, we examined the effectiveness and safety of erenumab in a sample of 140 French adult migraine sufferers included in the FHU InovPain regstry and who participated in a compassionate use program. According to the inclusion criteria of this compassionate use program, patients had at least 8 migraine days per month and previous failure of all available oral prophylactics whether approved (amitriptyline, flunarizine, metoprolol, oxetorone, pizotifen, propranolol, topiramate) or used off-label (candesartan, lisinopril, sodium valproate, venlafaxine).

In a per-protocol analysis considering patients who continued treatment up to each evaluation time, the results show that 52.9%, 58.5%, 57.0% and 58.8% of patients achieved ≥ 50% reduction in MMDs from baseline to month 3, month 6, month 9 and month 12 respectively. In an intention-to-treat (ITT) approach using LOCF, the results show that 52.9%, 50.0%, 46.4% and 46.4% of patients achieved ≥ 50% reduction in MMDs from baseline to month 3, month 6, month 9 and month 12 respectively. Such 50% response rates collected prospectively during one year of erenumab use were close to those estimated in a recent systematic review of real-world data related to CGRP-mAbs for migraine prophylaxis [9]. The per-protocol results show that 81.4%, 80.5%, 79.4% and 83.3% of patients achieved ≥ 30% reduction in MMDs from baseline to month 3, month 6, month 9 and month 12 respectively whereas ITT results show that 81.4%, 70.0%, 65.7% and 66.4% of patients achieved ≥ 30% reduction in MMDs from baseline to month 3, month 6, month and month 12 respectively. The 30% response rate is important to consider because 88.6% of the patients included in this real-world study suffered from CM at baseline and a 30% reduction in MMDs is suggested to be clinically meaningful for CM [16]. The benefit of erenumab for patients with CM is supported by the proportion of patients suffering from CM which was reduced to 37.9% at 3 months. The rate of reversion from CM to EM (57.3%) observed 3 months after the erenumab start in our real-world study is close to that estimated by a post-hoc analysis [17] of a randomized double-blind, placebo-controlled, phase 2 trial [18] and to those estimated in other real-world studies [12, 13]. This clinically relevant rate of reversion form CM to EM was associated with 46.5% of patients who presented a transition from MO to non-overuse at M3. The rate of transition from MO to non-overuse observed in our study is lower than that shown by a preplanned subgroup analysis [19] of the pivotal study that evaluated efficacy and safety of erenumab in patients with CM [18]. This difference may be due to the exclusion of patients using opioid-containing combination drug analgesics on ≥ 4 days/month in the pivotal study used for the subgroup analysis [18] whereas opioid overusers represented more than a quarter of the MO patients in our study.

For the whole population included in our study, the changes in MMDs, MHDs and HIT-6 score from M3 to M12 are close to those observed in the other prospective real-life studies that evaluated the efficacy of erenumab over at least one year [12–15]. Our logistic regression suggested that younger age was a positive predictor of erenumab effectiveness wheras high headache frequency and psychiatric comorbidity were negative predictors. A recent meta-analysis of real-world data identified a large number of positive predictors (good response to triptans, unilateral pain with, and unilateral autonomic symptoms) and negative predictors (obesity, interictal allodynia, the presence of daily headache, a higher number of previous prophylactic treatment failures, and psychiatric comorbidities including depression) [20]. Nevertheless, as discussed in a recent narrative review [21], the predictive value of these variables is not high and hence of low usefulness to select patients as candidates for CGRP-mAbs.

The adverse event incidence, the absence of serious adverse event, the low rate of treatment discontinuation due to adverse events and the most frequent adverse events observed in our study are close to those reported in erenumab randomized clinical trials [22] and their long-term extensions [23]. The main adverse event was constipation, as has been the case in other real-world studies of erenumab over a long period of time [12–15]. As in erenumab randomized clinical trials [22] there was no pattern of gastrointestinal history in patients who developed constipation in our study (data not shown). The proportion of patients who stopped treatment among those who experienced constipation (2/22) in our study was significantly lower than those observed in the two previous studies performed in Denmark [14] and in the UK [15]. Unlike the Danish and British studies, in which patients were seen every 3 months or less, our study enforced a monthly visit, making it easier to provide early treatment (dietary measures and/or symptomatic treatment), thereby reducing the discomfort caused by this adverse event. The results of systematic blood pressure measurement at each monthly visit for all patients in our study are reassuring. However, the HBP worsening in our patient with a HBP history and the patient with new-onset HBP reported in the UK study [15] indicate the importance of monitoring blood pressure in patients treated by CGRP-targeting drugs as confirmed in a recent Dutch study [24]. This real-world safety study performed on 196 Dutch patients treated with erenumab (109) or fremanezumab (87) in the Leiden Headache Center showed that the mean systolic and diastolic blood pressure increased after treatment with CGRP-mAbs was started with a long-lasting effect. The majority of patients remained within normal blood pressure limits, but 4 patients (3.7%) without HBP history required antihypertensive treatment after erenumab was started. Finally, one of our female patients became pregnant after 5 months of erenumab exposure without complications during pregnancy and no health issues in the new-born and in the post-partum mother. Pregnancy occurred in three female patients during the erenumab treatment and with a safe evolution after erenumab discontinuation in the UK study [15]. Such a good safety profile of CGRP-mAbs during pregnancy is suggested by an analysis of the World Health Organization pharmacovigilance database performed by Noseda et al. that found no specific maternal toxicicty, patterns of major birth defects or increased reporting of spontaneous abortion [25]. However, because of the relatively limited number of adverse drug reaction reported and the lack of long-term safety studies, CGRP-mAbs use should be avoided for migraine prevention in pregnant women. Further, the potential risk related to an unplanned pregnancy needs to be discussed with women of childbearing age before starting CGRP-mAb treatment [26]. The findings of the present study confirm, in real-world settings, the favorable benefit/risk of erenumab already supported by a ‘likehood of being helped or harmed’ analysis using data of erenumab randomized clinical trials [27]. Such a strong benefit/risk explains the high proportion of patients who reported being very or extremely improved.

This work is the first real-world study related to CGRP-mAbs use performed in France. It is unique as it involved the most difficult-to-treat patient population, since all the patients included had failed 11 oral prophylactic treatments. From a methodological point of view, the strengths of this study are as follows: the monthly prospective assessment, the duration of one year, the outcomes combining diary data and PROs such as HIT-6 and PGIC, the absence of patients lost to follow-up, and the combination of per-protocol analysis and an intention to treat analysis using LOCF approach. However, some study limitations need to be addressed. This study was monocentric and the generalization of its results on a national scale remains to be confirmed. Furthermore, although the patients could be considered ‘hopeless’ in terms of oral prophylaxis, they could not be considered ‘refractory’ according to the European Headache Federation criteria [28], since they had not been treated with Botox, which was not yet authorized in France at the time of their inclusion in our study.

## Conclusions

This first French real-word study related to CGRP-mAbs in migraine prevention confirms the effectiveness and safety of erenumab in patients with extreme unmet needs. We hope that such data, obtained in real-world settings but under satisfactory methodological conditions, will enable the negotiations between the French health authorities and the pharmaceutical companies to reach a positive issue, making this innovative therapeutic class available to the most severe migraine sufferers in France.

## Data Availability

The datasets used and/or analysis during the current study are available from the corresponding author on reasonable request.

## Abbreviations

AdjOR: Adjusted Odds-ratio
AVIESAN: National Alliance for Life Sciences and Health
CAV: Clinical added value
CGRP: Calcitonin gene-related peptide
CM: Chronic migraine
CNIL: Commission Nationale de l’Information et des Libertés
FHU: Federation Hospitalo-Universitaire
HFEM: High frequency episodic migraine
HBP: High blood pressure
ICHD-3: 3Rd edition of the International Classification of Headache Disorders
IQR: Median interquartile range
ITT: Intention-to-treat
LOCF: Last observation carried forward
mAbs: Monoclonal antibodies
MHD: Monthly number of headache days
MMD: Monthly number of migraine days
MO: Medication overuse
PGIC: Patient Global Impression of Change
SD: Standard deviation
STROBE: Strengthening the Reporting of Observational Studies in Epidemiology
95%CI: 95% Confidence interval
